# Adaptive representation of molecules and materials in Bayesian optimization†

**DOI:** 10.1039/d5sc00200a

**Published:** 2025-02-19

**Authors:** Mahyar Rajabi-Kochi, Negareh Mahboubi, Aseem Partap Singh Gill, Seyed Mohamad Moosavi

**Affiliations:** a Chemical Engineering & Applied Chemistry, University of Toronto Toronto Ontario M5S 3E5 Canada mohamad.moosavi@utoronto.ca; b Chemical & Materials Engineering, University of Alberta Alberta Canada

## Abstract

Bayesian optimization (BO) is increasingly used in molecular optimization and in guiding self-driving laboratories for automated materials discovery. A crucial aspect of BO is how molecules and materials are represented as feature vectors, where both the completeness and compactness of these representations can influence the efficiency of the optimization process. Traditionally, a fixed representation is chosen by expert chemists or applying data-driven feature selection methods on available labeled datasets. However, when dealing with novel optimization tasks, prior knowledge or large datasets are often unavailable, and relying on these even can introduce bias into the search process. In this work, we demonstrate a Feature Adaptive Bayesian Optimization (FABO) framework, which integrates feature selection in the Bayesian optimization process with Gaussian processes to dynamically adapt material representations throughout the optimization cycles. We demonstrate the effectiveness of this adaptive approach across several molecular optimization tasks, including the discovery of high-performing metal–organic frameworks (MOFs) in three distinct tasks, each involving unique property distributions and requiring a distinct representation. Our results show that the adaptive nature of the representation leads to outperforming random search baseline and scenarios where prior knowledge of the feature space is available. Notably, for known optimization tasks, FABO automatically identifies representations that are aligned with human chemical intuition, validating its utility for optimization tasks where such insights are not available in advance. Lastly, we show how a suboptimal representation, *e.g.*, when missing key features, can adversely impact BO performance, highlighting the importance of starting from a full feature set and adapt it to different tasks. Our findings highlight FABO as a robust approach for navigating large, complex materials search spaces in automated discovery campaigns.

## Abstract 2

Feature Adaptive Bayesian Optimization (FABO) enhances molecular and materials discovery by dynamically selecting optimal feature representations during Bayesian optimization, outperforming fixed representations.

## Graphical abstract

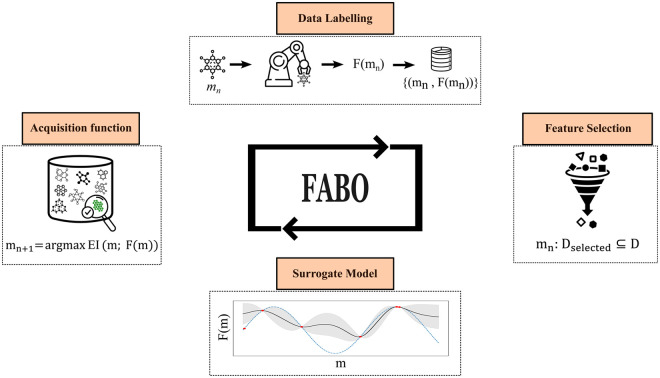

## Introduction

Recent advancements in machine learning (ML) and artificial intelligence (AI) are transforming molecular and materials discovery, driving the development of self-driving labs (SDLs) that integrate ML with lab automation and robotics.^1–4^ SDLs offer the potential to revolutionize research in chemistry and materials discovery by automating experimental workflows and enabling autonomous experimental planning. At the heart of SDL orchestration lies Bayesian optimization (BO), a framework that enables autonomous decision-making by balancing the exploration of new materials with the exploitation of existing knowledge, guiding the search toward optimal materials.^5–9^

A BO campaign starts with defining the search space, which involves converting materials and chemicals into numerical representations. Significant progress has been made in developing effective strategies to represent molecules for property prediction tasks, leading to the development of high-dimensional, complete representations with high learning capacity.^10–12^ However, in addition to the quality of the representation, the compactness is critical for BO performance.^13–15^ High-dimensional representation can lead to poor BO performance due to the curse of dimensionality. Previous research aimed to tackle this by tuning the surrogate model's receptive field through kernel length scale adjustments to facilitate high-dimensional BO.^16,17^ Another alternative is to use generative models to create embedding spaces for material representations.^18^ However, these methods often struggle to reconstruct materials when compressing information into lower-dimensional representations for BO, especially in advanced materials systems. As a result, current approaches tend to rely on expert intuition or data-driven feature selection methods based on labeled datasets. Yet, at the onset of materials discovery, the search space is completely uncharted and no labeled data are available. Generating labeled data to identify optimal features or representations would require additional experiments, using up precious resources on preliminary tests instead of allocating them to explore more materials during the Bayesian optimization process.

Metal–organic frameworks (MOFs) and related nanoporous materials exemplify the challenge of material representation in BO. MOFs are porous, crystalline materials with high tunable chemistry.^19^ Over the past two decades, more than one hundred thousand MOFs have been synthesized, and millions have been predicted *in silico*.^20–23^ Identifying the most promising MOFs for a given application is challenging using chemical intuition and traditional experimental methods. In this context, Deshwal *et al.*^24^ demonstrated the power of BO in identifying the best nanoporous materials for high-pressure methane storage applications, focusing on covalent organic frameworks (COFs). In such applications, pore geometry governs adsorption properties at high gas pressures. However, in other cases, a balance between pore geometry and materials chemistry, including the choice of metal and linker, determines material performance.^25,26^ Therefore, methods that can automatically adapt MOF representations in a BO campaign are essential for accelerating MOF discovery for diverse applications.

In this work, we introduce the Feature Adaptive Bayesian Optimization (FABO) framework, which systematically integrates feature selection into BO. FABO dynamically identifies the most informative features influencing material performance at each optimization cycle, enabling efficient BO for material discovery without prior representation knowledge. We benchmark FABO across multiple optimization tasks that require distinct representations, including: (1) MOF discovery across three case studies: CO_2_ adsorption at high and low pressures, and electronic band gap optimization; and (2) organic molecule discovery for water solubility and inhibition constant optimization. In all cases, FABO effectively reduces the dimensionality of the feature space and enhances the efficiency of BO, accelerating the identification of top-performing materials. Furthermore, by analyzing the automatically selected features, we demonstrate that they align with features a human expert might select for known tasks, showcasing FABO as a robust method for materials representation in novel optimization tasks where prior knowledge or data is lacking.

## Feature adaptive Bayesian optimization

**Fig. 1:**
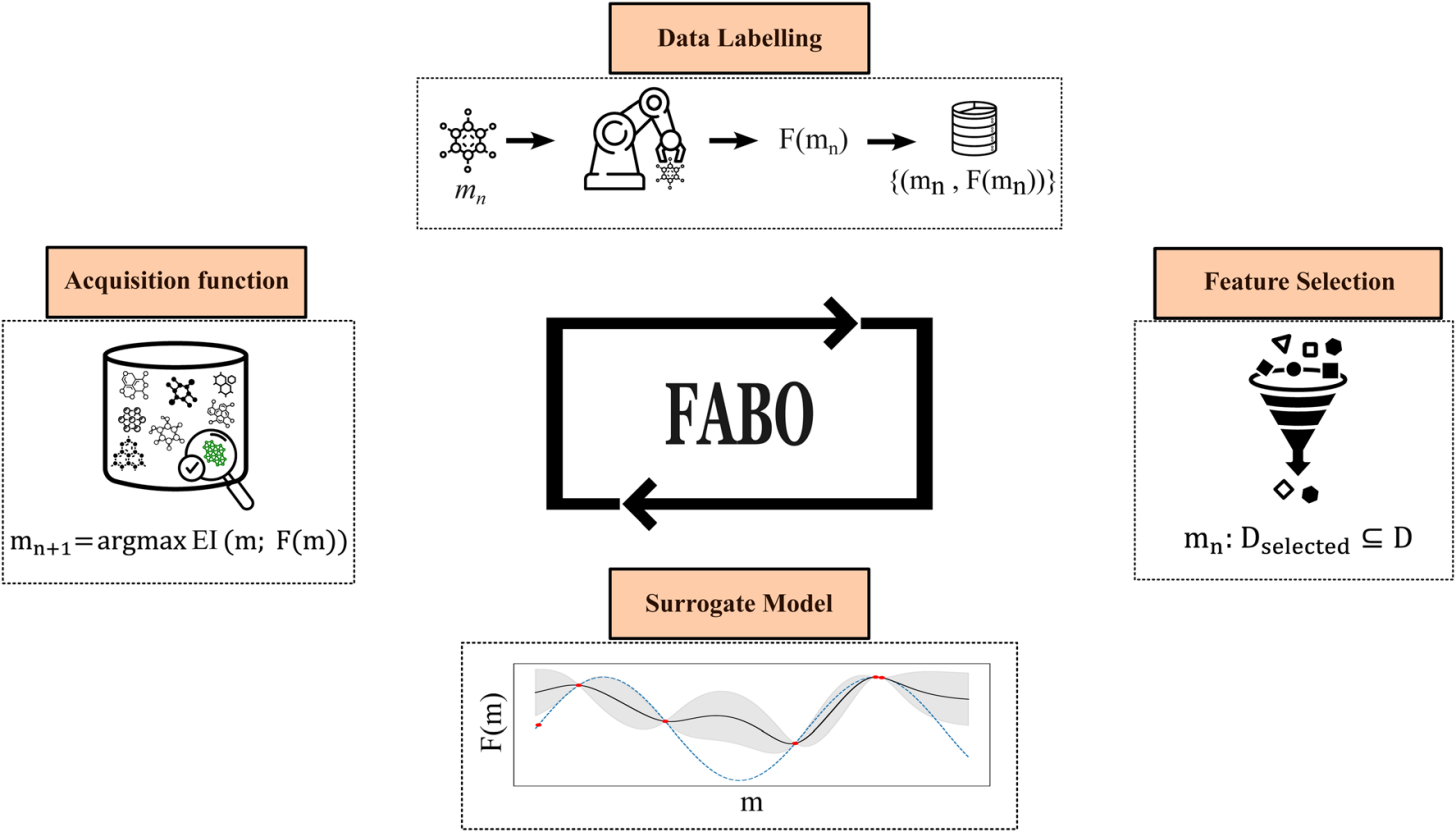
Feature Adaptive Bayesian Optimization (FABO) framework. FABO operates in an iterative feedback loop: (1) label the candidate material (*m*_*n*_) computationally or experimentally (*F*(*m*_*n*_)) and add it to the labeled dataset, (2) perform feature selection based on labeled data to determine the most informative representation, (3) update the surrogate model using the selected feature set (*D*_selected_), and (4) apply the acquisition function to select the next experiment (*m*_*n*+1_) for data labeling.

The workflow of Feature Adaptive Bayesian Optimization (FABO) is summarized in Fig. 1. The goal is to efficiently identify the best-performing materials from a large pool of candidates in a material database while minimizing the number of expensive experiments or simulations (*i.e.*, data labeling). Each closed-loop optimization cycle involves four key steps: data labeling, updating materials representation, updating the surrogate model, and selecting the next experiment to perform using an acquisition function.

BO relies on two core components for decision-making: a predictive surrogate model that estimates the objective function with uncertainty quantification, and an acquisition function that guides the selection of the next material to sample.^27^ The acquisition function balances exploitation (choosing materials for which the model predicts optimal values) with exploration (sampling areas of high uncertainty to gather new information).^28,29^ In this study, we employ a Gaussian Process Regressor (GPR) as the surrogate model due to its strong uncertainty quantification capabilities, and two acquisition functions, namely the Expected Improvement (EI) and Upper Confidence Bound (UCB), which are popular choices in BO.^30^

The input to the surrogate model is a numerical representation of the materials. Since the decision-making in BO depends on the previous evaluations but is invariant to their order, we can treat each optimization step as an independent BO cycle and adapt the material representation at each cycle. Rather than relying on a fixed, predefined feature set or requiring a large amount of labeled data upfront for feature selection, we start with a complete, high-dimensional material representation, and at each optimization cycle, we refine this representation using feature selection methods to identify the most relevant features. In this case, we only use the acquired data during the BO campaign for the feature selection. This enables autonomous exploration of the search space with minimal prior information about the best representation.

We investigate two feature selection methods in this study; however, any feature selection method can be incorporated into the feature selection module of FABO. The first method, Maximum Relevancy Minimum Redundancy (mRMR), selects features by balancing relevance to the target variable *y* and redundancy with respect to the already selected features ({*d*_*j*_, *d*_*k*_, …}). For a given candidate feature *d*_*i*_, the mRMR score is computed as:1
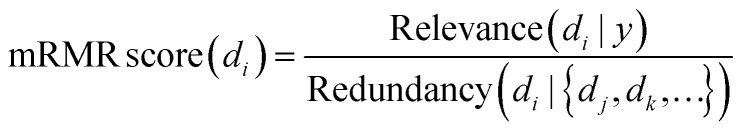


Relevance measures how strongly the candidate feature *d*_*i*_ is related to the target *y*. This is calculated using the F-statistic, which quantifies the statistical relationship between the feature and the target. A higher relevance value indicates that the feature has significant explanatory power for *y*. Redundancy represents the average correlation of the candidate feature *d*_*i*_ with the already selected features ({*d*_*j*_, *d*_*k*_, …}). By minimizing redundancy, the algorithm ensures that newly selected features add unique and non-overlapping information. Initially, the first two features are selected purely based on their relevance to the target. Subsequent the algorithm iteratively selects features by maximizing the mRMR score for each candidate feature *d*_*i*_, continuing until the desired number of features is selected.^31^ To implement this process, we use the mRMR Python package.^32^

The second method we utilize is Spearman ranking, a univariate, ranking-based technique. It evaluates each feature based on its Spearman rank correlation coefficient with the target variable, measuring the strength and direction of the monotonic relationship between the two. Both of these methods are computationally efficient and easy to implement, making them well-suited for iterative optimization processes like BO.^33,34^ In our BO runs, we select between 5 and 40 features for CO_2_ uptake, and between 5 and 20 for band gap optimization, from the feature pool using these selection methods. Detailed information about the feature selection methods and the full workflow can be found in the ESI.†

## Case studies

We focus our case studies in this section on the discovery of MOFs with specific target properties from large databases. More benchmarking on molecular properties, such as molecular solubility, can be found in ESI materials.† MOFs are an ideal test case for the FABO framework due to the complex relationship between geometry and chemistry that heavily influences their properties. This complexity makes identifying optimal representations for Bayesian optimization especially challenging. In this study, we utilize two key datasets: (1) the QMOF database including 8437 materials with the electronic band gaps of MOFs calculated using high-throughput periodic density functional theory (DFT),^35,36^ and (2) the gas adsorption properties for Computational Ready MOF database (CoRE-2019)^37^ with 9525 materials, for which we took CO_2_ adsorption data at low (0.15 bar) and high pressures (16 bar) at room temperature from a previous study.^26^ Given our prior knowledge of how both chemistry and geometry affect these properties from previous works,^25,26,35^ it provides the opportunity to compare the representations adapted by FABO to those following our chemical intuition. Specifically, the band gap is largely influenced by the material's chemistry,^35^ gas uptake at high pressure is primarily determined by geometry, and gas uptake at low pressure is influenced by a combination of both chemistry and geometry.^26^

We begin with a complete representation of each MOF, where the pool of features includes both chemical and pore geometric characteristics. To represent chemistry of the materials, we use Revised Autocorrelation Calculations (RACs)^26,38,39^ alongside two stoichiometric feature sets. RACs capture the chemical nature of MOFs by relating heuristic atomic properties, such as electronegativity and nuclear charge, across atoms in a graph representation of the material. As RACs are computed over the crystal graph of the material, they contain bond geometric information in addition to pure chemical features. This set of descriptors is augmented by heuristics like ionization energy, electron affinity, and atomic group and row numbers. In addition, we include two stoichiometric feature sets: stoichiometric-45, developed by He *et al.*, which includes 45 elemental property descriptors,^40^ and Stoichiometric-120, which contains 103 elemental fraction descriptors and 17 statistical attributes.^41^ Both are based entirely on the chemical composition of the materials. Moreover, for features describing the pore geometry, we use eight descriptors, including the largest included sphere (*D*_i_), largest free sphere (*D*_f_), the largest included sphere along the free path (*D*_if_), crystal density (*ρ*), as well as volumetric and gravimetric surface areas and pore volumes, calculated using Zeo++.^42^ Previous studies have demonstrated that combining these chemical and geometric features is sufficient to train machine learning models capable of predicting both band gap and gas uptake at low and high pressures, making this feature set a robust starting representation for our case studies.^26,35^

While this high-dimensional feature vector is expressive for property regression tasks, using such a high-dimensional representation in BO can significantly reduce efficiency. Therefore, selecting a smaller, more informative set of features is necessary. We explore different scenarios for feature selection and compare their performance to FABO's automatic, adaptive feature selection process. The first scenario involves feature selection guided by expert intuition, where chemists choose features deemed most relevant to the optimization task. The second scenario assumes the existence of a fully labeled dataset for the property of interest, allowing for traditional feature selection before the BO process begins. While this approach can yield highly effective representations, it is often impractical in early-stage materials discovery, where only a limited number of experimental measurements are available. In another scenario, labeled data for a related property is used as a proxy for feature selection, and the selected features are transferred to a similar, though not identical, optimization task (*e.g.*, using partial charge data to optimize band gap). In contrast to FABO, all of these methods rely on fixed feature sets that do not change throughout the BO campaign. Once feature selection is performed, the feature set remains static, even as new data is acquired. FABO, however, dynamically updates the feature representation as new labeled data becomes available, continuously refining the search process.

**Fig. 2:**
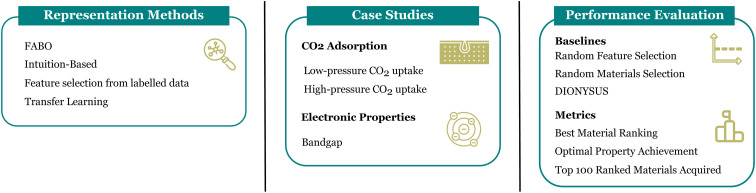
Overview of the case studies in this study this includes material representation, the datasets, and performance evaluation.

Finally, we compare the performance of these methods to three baselines: (1) random feature selection for BO, and (2) random material selection, where materials are chosen at random without the guidance of BO, and (3) DIONYSUS,^17^ a BO framework which uses the full feature set and adjust the kernel length scale for each feature throughout the BO process. A summary of feature selection methods, the performance evaluation, and datasets are shown in Fig. 2.

In our benchmarking, the BO cycles, including the cycle of planning (*i.e.*, selecting the next point) and inference (updating the model with new observations) is repeated for up to 250 iterations, assuming a budget of 250 experiments/computations to find the best material. As the initial data points can lead to uncertainty in Bayesian optimization campaigns, we run 20 independent BO campaigns, each with 10 different initial data points randomly selected from the original pool. This approach mitigates the risk of relying on a single, potentially unrepresentative starting set. If BO campaigns are conducted with only one set of initial data points, the optimization process becomes overly dependent on that particular set, leading to biased results and potentially missing the true optimal solution.^43^

### Performance evaluation of FABO

**Fig. 3:**
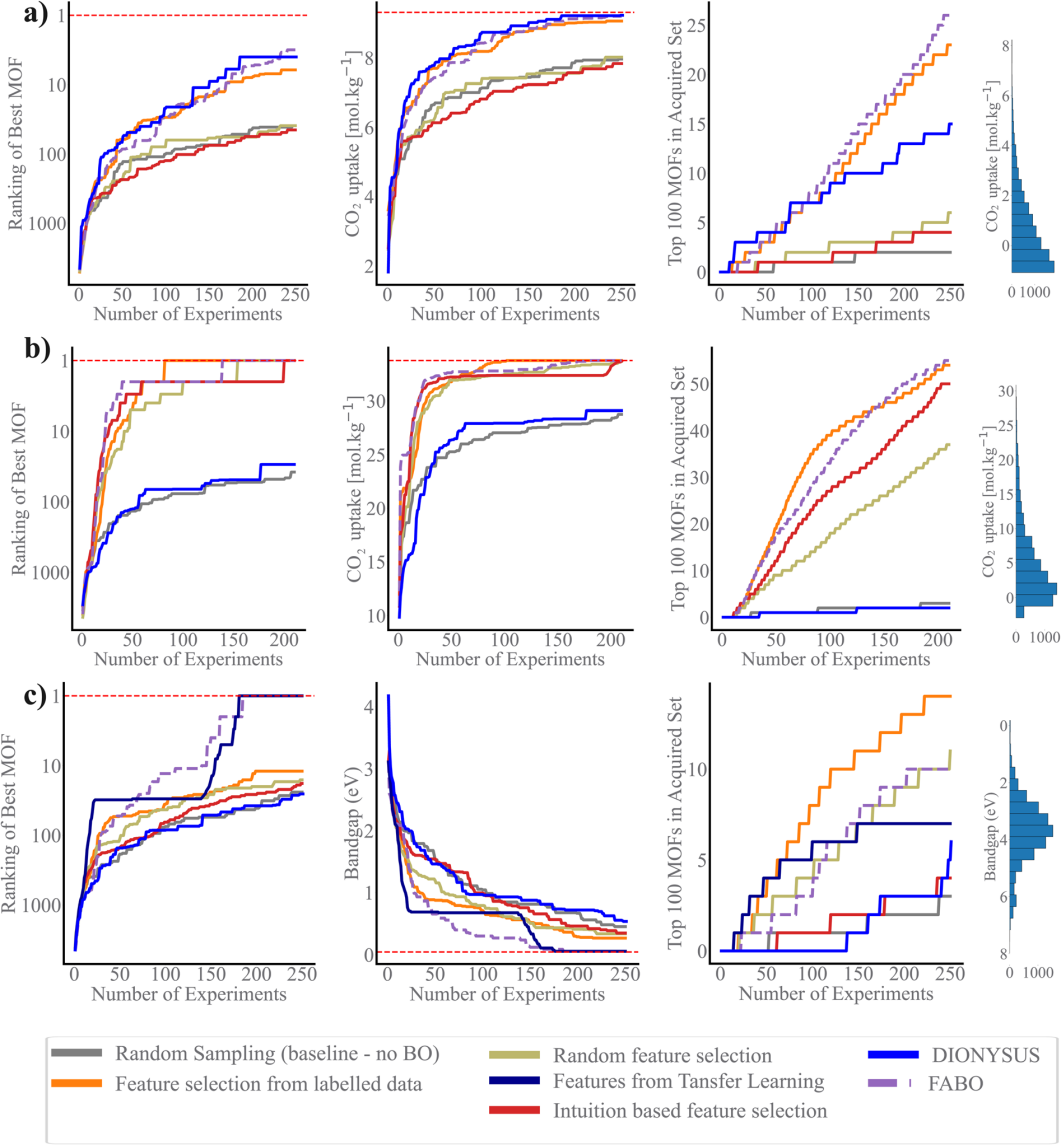
Search efficiency curves for different representation methods. Results represent the average values across 20 trials for each method, with three performance metrics: the best rank, the best value, and the number of acquired materials among the top 100 materials in the dataset. (a) CO_2_ uptake at low pressure, (b) CO_2_ uptake at high pressure, and (c) band gap.

We use three metrics to evaluate the quality of the acquired MOFs during the BO campaign: the best rank, the best value of the objective function, and the number of acquired materials among the top 100 materials in the dataset.^44^ The search efficiency curves in Fig. 3 demonstrate the high performance of FABO across all three metrics and three objectives. FABO consistently outperforms the baselines, and shows performance similar to the cases where we have prior knowledge *via* expert intuition or labeled dataset. The superior performance of FABO compared to random search clearly highlights the power of BO in materials optimization and discovery, surpassing traditional trial-and-error approaches. Additionally, FABO's outperformance of random feature selection underscores the critical role of selecting appropriate representations for different BO tasks.

Remarkably, FABO performs similarly or better than BO campaigns that use fixed features obtained from feature selection methods applied to labeled datasets. Feature selection methods typically rely on labeled datasets to identify features that have the strongest relationship with the target property. However, in the early stages of material discovery, labeled datasets are unavailable. In a hypothetical scenario, let us assume a labeled dataset exists. In this case, we apply two machine learning-based feature selection methods—Spearman ranking and mRMR—to select the top 5 and 40 features. We then run BO campaigns using these fixed, pre-selected features to evaluate performance. This serves as a benchmarking model, simulating the availability of a fully labeled dataset.

However, FABO excels by overcoming the limitations of such static approaches. On one hand, it does not require any pre-labeled data; it starts from scratch, dynamically acquiring labeled data by prioritizing materials likely to have distinguished properties (exploitation aspect of BO) while simultaneously adapting the features to the labeled materials acquired so far during the optimization process. On the other hand, it demonstrates similar or better performance compared to BO campaigns that use fixed features. For instance, in the low-pressure CO_2_ uptake task, BO using a fixed feature set obtained through Spearman ranking—applied to over 9500 MOFs—fails to capture even 10% of the top 100 MOFs (Fig. S2 in ESI†). In contrast, adapting the feature set with BO throughout the optimization process results in identifying over 20% of the top 100 MOFs (Fig. 3a). In the high-pressure CO_2_ uptake and band gap optimization tasks, all BO campaigns, whether or not they have access to label-annotated data, successfully identify the optimal solution, though some converge more quickly than others (Fig. S2 in ESI†). The key distinction, however, lies in the fact that FABO follows a more practical approach. It successfully identifies the highest-ranking MOF by the 135th iteration, nonetheless, relies solely on the available knowledge of the search space and operates without any prerequisite information (Fig. 3b). This makes FABO better suited for real-world scenarios where labeled data is often scarce or unavailable at the outset.

While incorporating expert knowledge in BO (*i.e.*, intuition-based feature selection) offers more strategic guidance than random feature selection, the results in Fig. 3 show intuition-based feature selection often falls short in fully capturing the complexity of structure–property relationship. In specific, for the more complex properties, namely CO_2_ uptake at low pressure and band gap, which involve complex chemistry, intuition could not identify the best features for the tasks. For these two tasks, the performance of BO using intuition-based representation is as poor as random selection. On the other hand, for the simpler problem of high-pressure CO_2_ uptake, which requires only geometric features, mainly pore volume, the intuition based feature selection successfully identifies the best MOFs (Fig. 3b). These outcomes suggest that while intuition-based methods may be advantageous in specific scenarios, they fail to fully leverage the dataset's richness and complexity, limiting their effectiveness across different tasks and even can introduce bias to the search.

Transfer feature selection can be an effective way for feature selection. This approach leverages knowledge from similar optimization tasks, making it particularly useful when data from a related domain is available or inexpensive to obtain. In our case study on optimizing band gap, we utilize features that are most informative for predicting the partial charge of MOFs, as both properties are influenced by the material's electronic structure. By engineering features based on labeled partial charge data and applying them to represent MOFs for band gap optimization, we achieve significantly better performance compared to random search and random feature selection. However, the fact that FABO outperforms transfer feature selection highlights its effectiveness in scenarios where no prior information is available, underscoring its robustness for discovery tasks with limited or no existing data.

Previous research has suggested that BO using a Gaussian process (GP) as the surrogate model struggles with efficiency in high-dimensional spaces due to the curse of dimensionality, specifically when a single kernel length is used for all dimensions. A remedy for this is to tune the length scale for each feature such that the features with large length scales become less important, akin to “feature deselection”, whereas features with smaller length-scales are treated as highly relevant for predictions. DIONYSUS^17^ is a GP model that follows this, in which it employs a squared exponential (RBF) kernel with automatic relevance determination (ARD), allowing each feature to have its own length-scale parameter. These length-scales are optimized during training *via* gradient-based methods. Fig. 3 shows that while DIONYSUS can be effective and shows similar performance to FABO in some scenarios, namely CO_2_ uptake at low pressure, it struggles in cases where uninformative features dominate the input space, as seen in our experiments with CO_2_ uptake at high pressure and band gap optimization. The key difference lies in how each method handles irrelevant features. FABO explicitly eliminates these features by assigning them a zero weight, whereas DIONYSUS retains them with very large length scales, which can still introduce noise into the model. For CO_2_ uptake at high pressure, where only a few features are informative, FABO's ability to completely exclude irrelevant features enhances optimization robustness.

Random forests are well-known for their ability to perform automatic feature selection by assigning importance scores to features, making them a potentially valuable surrogate model in Bayesian optimization. To evaluate their performance, we implement BO campaigns using a random forest surrogate model instead of GP. In this setup, the mean prediction is obtained by averaging outputs from individual trees, and the uncertainty is estimated from the variance of these predictions. The results, as shown in Fig. S4,† indicate that while BO with random forests can be effective in CO_2_ uptake at low pressure, it struggles with tasks like CO_2_ uptake at high pressure and band gap optimization and fails to converge to the material with optimal properties within 250 iterations. The challenges stem from the limitations of random forests in accurately quantifying uncertainty, particularly outside the coverage of the training set. Unlike GPs, which provide smooth and continuous predictive surfaces with well-calibrated uncertainty estimates, random forests rely on ensemble variance, which can be noisy and unreliable for guiding exploration.

**Fig. 4:**
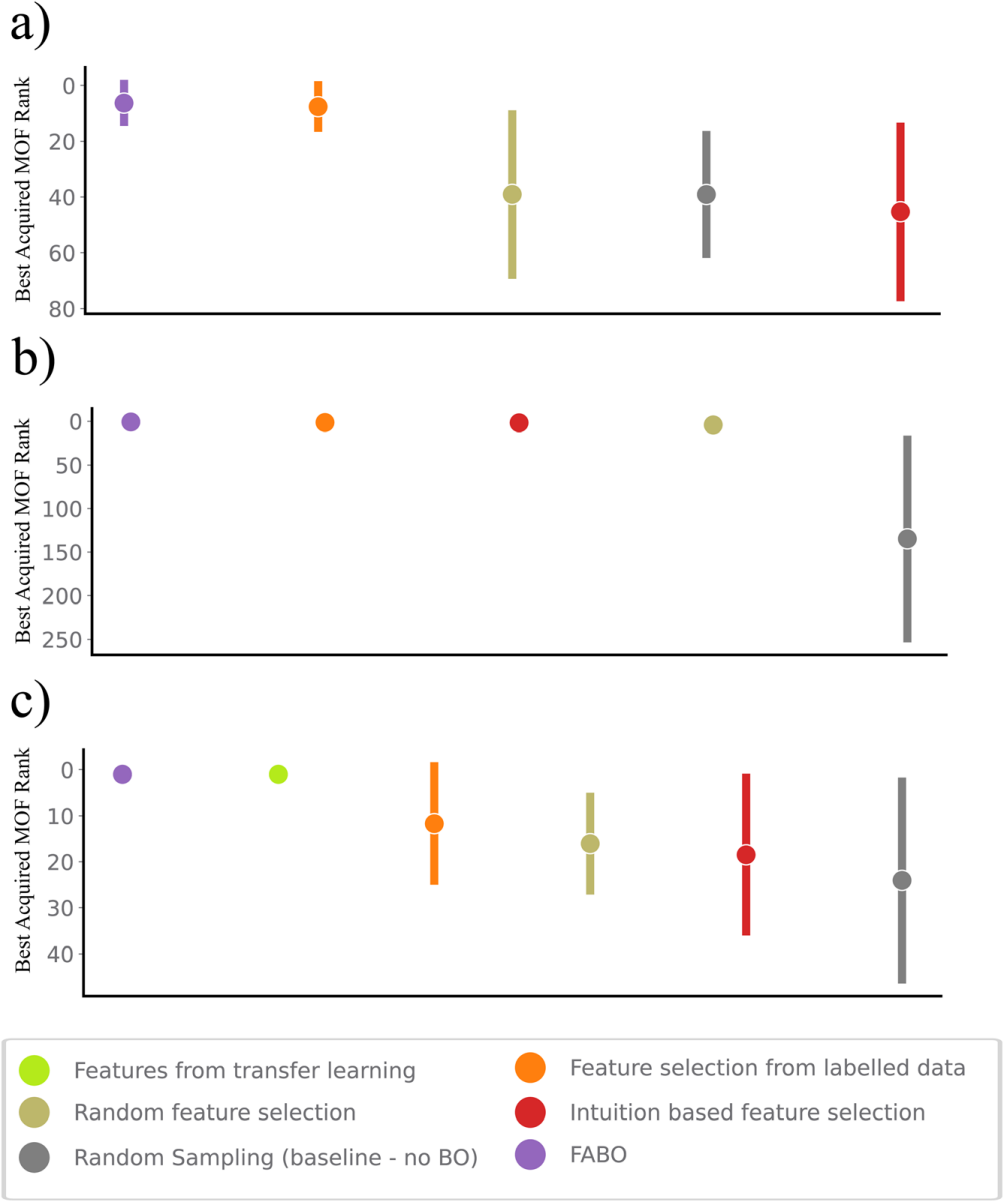
Uncertainty analysis for the three optimization tasks. The error bars illustrate the standard deviation of the rankings of the best-acquired MOFs across 20 trials, calculated after 250 iterations for each method. This iteration represents the assumed experimental budget, enabling a meaningful comparison of the models' performance under the same constraints. (a) CO_2_ uptake at low pressure, (b) CO_2_ uptake at high pressure, and (c) band gap. FABO is compared against baseline models and fixed-feature Bayesian optimization approaches.

In sequential design strategies, the starting point of the searching process plays a crucial role, as it can significantly influence the primary knowledge of surrogate model and can result in getting stuck in local minima. To assess the impact of initial points on BO performance and the resulting uncertainty in identifying the highest-ranked materials, we plot the best rank after 250 cycles across various methods (Fig. 4). Notably, FABO identifies the best materials in 250, 135 and 170 number of iterations for CO_2_ low pressure, high pressure and band gap, respectively. Integrating adaptive feature selection with BO minimizes the uncertainty associated with the campaign starting points, making the algorithm become independent of the initial condition, and more stable and reliable.

Finally, we note that the choice of feature selection method can influence FABO's effectiveness. In particular, the mRMR method performs better than Spearman correlation-based approaches. This improvement is largely due to the mRMR's ability to eliminate redundant features, leading to a more compact and informative representation, which Spearman correlation does not effectively address. Moreover, while FABO effectively guides the optimization process, tuning hyperparameters across various components of the model remains essential, particularly in complex tasks where finding the best candidate can be challenging. For example, in the band gap minimization, fine-tuning the acquisition function demonstrates a significant impact on performance. By switching from expected improvement to upper confidence bound for the first 100 iterations, FABO enhances exploration and reduces model uncertainty, focusing on active learning. After the initial exploration phase, reverting to EI enables the model to better exploit the learned patterns, ultimately leading to improved performance. This hybrid acquisition strategy allows FABO to converge to the top-ranked MOF in the band gap minimization task after 170 iterations, significantly enhancing the optimization outcome (Fig. S3 in ESI†).

### Understanding the adapted representation

**Fig. 5:**
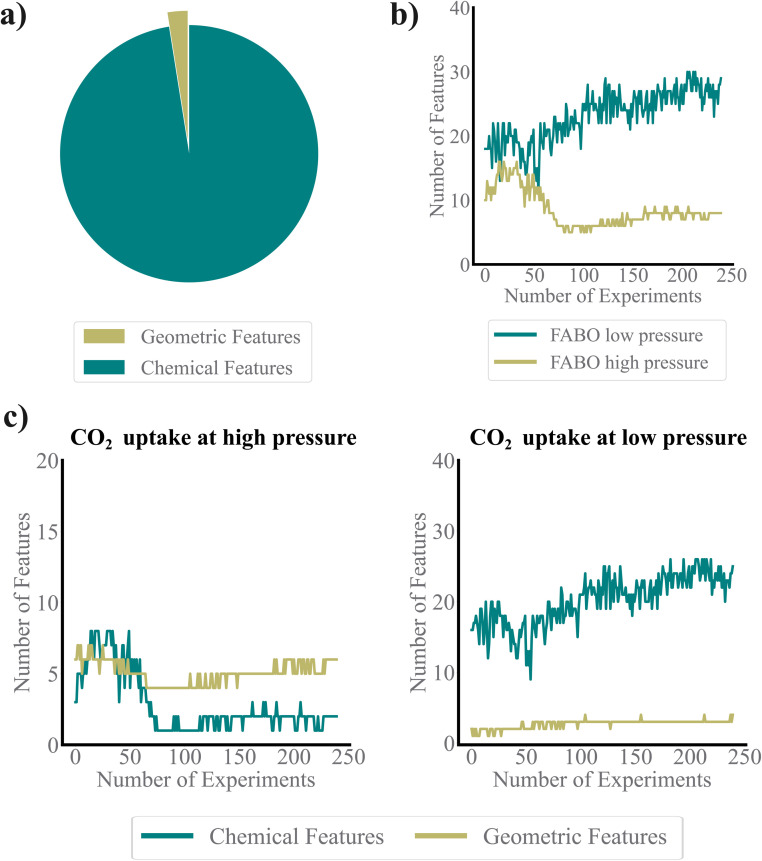
Understanding adapted representation throughout the BO cycles. (a) Distribution of geometric and chemical features within the dataset's feature pool. (b) Feature set size during FABO optimization for identifying MOFs with the highest CO_2_ uptake at both low and high pressures. (c) Number of chemical and geometric features utilized by FABO to represent MOFs at high and low pressure.

Monitoring the features selected by FABO provides valuable insights into whether its choices align with expert's chemical intuition. The feature pool used in this study consists of two main sets: geometric and chemical features, each capturing distinct aspects of MOF structure. Chemical descriptors dominate the pool, making up the majority of features, while geometric features account for only about 2.5% of the total (Fig. 5a). From previous works, we know that adsorption is driven by geometric features at high pressure, due to the physical confinement and the available pore volume within the MOF structures. In contrast, at low pressures, subtle chemical interactions between the adsorbate and the MOF makes chemical features critical for accurately predicting performance.^26^

Fig. 5c shows that the representation adapted by FABO for each task follows these chemical understandings. For high-pressure CO_2_ uptake, geometric features dominate the representation, which reflects their importance in predicting adsorption properties at high pressures. Conversely, for low-pressure CO_2_ uptake, chemical features consistently constitute the majority of features. Notably, while chemical features remain predominant in low-pressure conditions, FABO effectively adapts to capture the growing importance of geometric features as the optimization progresses, adjusting its representation to enhance the search process.

We observe a significant difference in the number of selected features between the low- and high-pressure cases. In the low-pressure case, a larger number of features are selected, indicating that a broader range of descriptors becomes relevant as the model explores the search space (Fig. 5b). This suggests that optimizing for low-pressure CO_2_ uptake requires capturing a wider variety of characteristics, reflecting the complexity of the property. In contrast, the high-pressure case sees a decreasing number of selected features, implying that a more specific and refined set of descriptors is sufficient to predict the target property accurately. This divergence underscores the different nature of the two tasks: for low-pressure adsorption, the feature set is more complex, whereas for high-pressure adsorption, the model converges to a simpler, low-dimensional feature set. Over time, however, as more labeled data become available through FABO, the feature sets for both tasks stabilize and reach a plateau, indicating that further data do not alter the selected feature set. Interestingly, FABO does not start with a large feature set at the beginning of the BO process due to the limited amount of available data. This follows the bias-variance trade-off, giving FABO a distinct advantage over fixed representations, even those selected from labeled datasets. FABO selects the most appropriate features based on the current dataset, dynamically adjusting as new data is acquired. Moreover, this reduction in the number of features for the high-pressure task also explains why intuition-based feature selection may work better in simpler cases. Since this problem is lower dimensional, a human expert can more easily conceptualize the key features, making manual feature selection more effective.

### Influence of suboptimal representation on BO

It is interesting to investigate how BO performs when starting with a suboptimal representation—a feature set that fails to capture the material characteristics most relevant to the property of interest. Such a scenario can arise when a human manually selects features based on incomplete knowledge or assumptions, inadvertently excluding essential descriptors, and biasing the search. To mimic this situation, we designed experiments where Bayesian optimization was tested with two deliberately restricted feature pools: one consisting solely of geometric descriptors and the other containing only chemical descriptors.

**Fig. 6:**
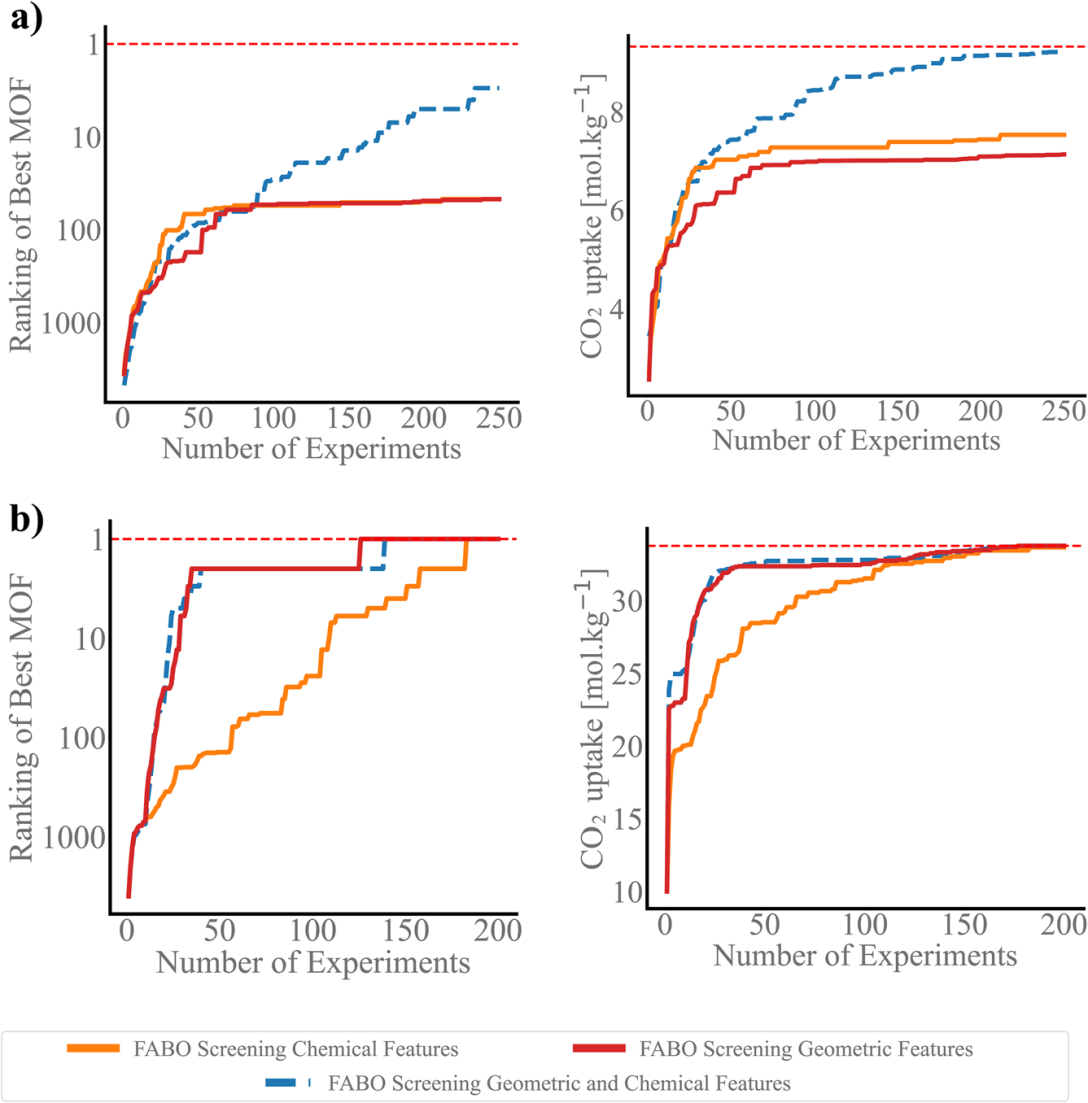
Impact of suboptimal representation on BO. Each plot compares the best rank achieved (left) and the highest actual CO_2_ uptake obtained (right) when FABO uses only geometric features, only chemical features, and when dynamically selecting features from both categories, for (a) low-pressure CO_2_ uptake and (b) high-pressure CO_2_ uptake.

Fig. 6 clearly demonstrates that when the BO model is constrained by limited feature information, its ability to identify the optimal MOF is significantly impacted. For example, in the low-pressure CO_2_ adsorption task, running BO on a suboptimal feature set with only a specific type of features results in the selection of a MOF with a CO_2_ uptake of 7.25 mol kg^−1^, which is more than 28% lower than the maximum CO_2_ uptake achieved by a MOF in the full dataset. The lack of balanced feature representation limits the model's capacity to capture the underlying complexity of MOF behavior in this context. In contrast, FABO, which has access to the full feature pool containing both chemical and geometric features, outperforms models that rely on suboptimal feature sets demonstrating the importance of both types of descriptors in the optimization process, as each contributes unique and essential information (Fig. 6a). The trade-off between chemical and geometric descriptors becomes clear when one set is omitted, leading to suboptimal search outcomes. Interestingly, in the high-pressure CO_2_ uptake task, a model using only geometric features performs significantly better, identifying the top-ranked MOF 50 iterations earlier than a model relying solely on chemical features. However, FABO still matches the performance of geometric-only model, highlighting its capacity to detect when certain feature classes (in this case, chemical features) are less critical. This adaptability prevents this approach from overloading the optimization process with irrelevant descriptors, streamlining the search while maintaining high performance.

## Conclusion

In this work, we introduced the Feature Adaptive Bayesian Optimization (FABO) framework, which integrates feature selection into Bayesian optimization to dynamically refine material representations throughout the optimization process. Our approach addresses a key challenge in materials discovery: identifying effective representations for complex materials, such as MOFs, in the absence of labeled data or prior knowledge. FABO offers a more flexible and efficient solution compared to traditional methods that rely on static, fixed feature sets.

Through a series of case studies, including low- and high-pressure CO_2_ uptake and band gap optimization, we demonstrated that FABO consistently outperforms or matches feature selection approaches based on labeled data. Its ability to adapt feature sets during the optimization allows it to capture the evolving importance of different descriptors—such as geometric features in high-pressure tasks and chemical features in low-pressure tasks—overcoming the limitations of fixed representations. This adaptability enhances the search process and positions FABO as a superior tool for real-world discovery scenarios, particularly where annotated data is scarce or unavailable at the outset.

Finally, our open-source implementation of FABO is available for researchers to easily apply to their own domain-specific optimization problems. By starting from a complete feature set, FABO's integrated feature selection within BO ensures that the most relevant features are dynamically chosen to optimize the search space efficiently.

## Data availability

The code base for FABO as well as the codes and data to reproduce results of this study are available from https://github.com/AI4ChemS/FABO.

## Author contributions

Conceptualization: S. M. M., M. R. K., N. M., A. P. S. G.; data curation: M. R. K., N. M., A. P. S. G.; formal analysis: M. R. K., N. M., A. P. S. G.; funding acquisition: S. M. M.; investigation: M. R. K., N. M., A. P. S. G.; methodology: M. R. K., N. M., A. P. S. G., S. M. M.; project administration: S. M. M.; software: M. R. K., N. M., A. P. S. G.; supervision: S. M. M.; visualization: M. R. K., N. M., A. P. S. G.; writing: M. R. K., N. M., & S. M. M.

## Conflicts of interest

The authors declare no competing interests.

## Supplementary Material

SC-016-D5SC00200A-s001
