# Supplementary material for: Adaptive representation of molecules and materials in Bayesian optimization
Source: Chem Sci. 2025 Feb 19;16(13):5464–74. doi: 10.1039/d5sc00200a (PMC11851078; doi:10.1039/d5sc00200a)
Supplement: SC-016-D5SC00200A-s001 [file SC-016-D5SC00200A-s001.pdf]

Supplementary Information (SI) for Chemical Science.
This journal is © The Royal Society of Chemistry 2025

Supplementary Materials for Adaptive Representation of Molecules and Materials in Bayesian Optimization

Mahyar Rajabi Kochi,[†,¶] Negareh Mahboubi,[‡,¶] Aseem Partap Singh Gill,[†] and Seyed Mohamad Moosavi[*,†]

†*Chemical Engineering & Applied Chemistry, University of Toronto, Toronto, Ontario M5S 3E5, Canada*

‡*Department of Chemical and Materials Engineering, University of Alberta, Alberta, Canada*

¶*Contributed equally to this work*

E-mail: mohamad.moosavi@utoronto.ca

Contents

1 Mechanism and Workflow of FABO **3**

1.1 Initialization . . . 3

1.2 Feature engineering . . . 3

1.3 Surrogate Model . . . 5

1.4 Acquisition function . . . 5

1.5 Labeling . . . 6

2 Further Analysis on FABO **8**

2.1 BO with Random Feature Selection . . . 8

2.2 BO with Features Selected from Labeled Dataset 9
2.3 FABO with Different Feature Selection Method 10
2.4 Benchmarking FABO against BO with random forest as surrogate model . . 11
2.5 Random Embedding Bayesian Optimization (REMBO) 12
2.6 Benchmarking FABO on Molecular Datasets: CHEMBL and Delaney 14
2.7 Comparative Summary of Material Representation Approaches in Bayesian optimization 15
2.8 Benchmarking FABO on Molecular Datasets: CHEMBL and Delaney 16

3 Compute resources **17**

References **17**

1 Mechanism and Workflow of FABO

In a comprehensive search, costly simulations and experiments would be required to determine the properties of interest for each candidate material in the dataset M, generating pairs $\{(m, F(m)) : m \in M\}$ to identify the optimal material (m^*) with the desired characteristics. However, rather than conducting this exhaustive approach, our goal is to efficiently pinpoint the m^* while performing these expensive simulations or experiments on only a small subset of candidates. Bayesian optimization optimizes decision making concerning which candidate needs to be set next for iteration.

1.1 Initialization

We initialize BO campaign with 10 initial materials selected randomly from the original pool $M = \{(m_i, F(m_i)) \mid i = 1, 2, \dots, N\}$ without replacement, then they are labelled. This process is repeated 20 times, with the selected materials added to the selected points collection $M_s = \{(m_i, F(m_i)) \mid i = 1, 2, \dots, 10\}$. Repeating the initial selection stage 20 times accounts for the uncertainty in the dataset and its influence on the search direction in Bayesian optimization. Multiple initialization helps reduce the risk of the optimization process becoming overly dependent on a single, potentially unrepresentative starting set.

1.2 Feature engineering

To proceed with the Bayesian optimization, each material structure must be represented as a vector, composed of structural and chemical features. We integrate two feature selection methods into the BO loop to identify the most important features from the prior labeled dataset (M_s). The feature set size can vary between 5 and 40, aiming to find the optimal set of features (D_{selected}) from the feature pool (D). The first method, Maximum Relevancy Minimum Redundancy (mRMR), selects features by balancing relevance to the target variable y and redundancy with respect to the already selected features ($\{d_j, d_k, \dots\}$). For a

given candidate feature d_i, the mRMR score is computed as:

$$\text{mRMR Score}(d_i) = \frac{\text{Relevance}(d_i \mid y)}{\text{Redundancy}(d_i \mid \{d_j, d_k, \dots\})} \tag{1}$$

Relevance measures how strongly the candidate feature d_i is related to the target y. This is calculated using the F-statistic, which quantifies the statistical relationship between the feature and the target. A higher relevance value indicates that the feature has significant explanatory power for y. Redundancy represents the average correlation of the candidate feature d_i with the already selected features ($\{d_j, d_k, \dots\}$). By minimizing redundancy, the algorithm ensures that newly selected features add unique and non-overlapping information. Initially, the first two features are selected purely based on their relevance to the target. Subsequent the algorithm iteratively selects features by maximizing the mRMR score for each candidate feature d_i, continuing until the desired number of features is selected. To implement this process, we use the mrmr Python package.

The second method, Spearman ranking, is a univariate, ranking-based method. It evaluates each feature based on its Spearman rank correlation coefficient ρ with the target variable. Spearman's rank correlation measures the strength and direction of the monotonic relationship between two variables. The Spearman correlation coefficient for a feature d_i is calculated as:

$$\rho(d_i, y) = 1 - \frac{6 \sum_{i=1}^{n} \gamma_i^2}{n(n^2 - 1)} \tag{2}$$

where γ_i represents the difference between the ranks of d_i and y, and n denotes the number of observations. Features are ranked by the magnitude of their correlation coefficients, with the top-ranking features being selected.

1.3 Surrogate Model

Gaussian process regressor (GPR) which models the objective function ($F(m)$) as a distribution over possible functions, is used as surrogate model of FABO. The model is defined by a mean function and a covariance function (kernel), which control the behavior and smoothness of the function. A GPR approximates the relationship between input features and the target variable and provides uncertainty estimates. Given a set of featurized materials (M_s) and corresponding outputs (y), the GP computes the posterior distribution at any new point based on the prior distribution and observed data. This enables predictions on unseen points while providing uncertainty estimates, which are crucial for determining the next sampling point in Bayesian optimization.

1.4 Acquisition function

The next critical step in Bayesian optimization is determining the next sampling location based on the inferences drawn from the fitted model. This is achieved using acquisition functions, which assess the potential value of information gained by sampling at a specific point. For deterministic responses, the Expected Improvement (EI) acquisition function is one of the most commonly used methods and performs well across various problems. EI strikes a balance between exploitation (choosing points where the model predicts optimal values) and exploration (sampling in areas of high uncertainty). In our context, the GP model is first fitted to the labeled materials, with the fitted mean prediction $\hat{y}(m)$. Instead of selecting the point that optimizes $\hat{y}(m)$, EI incorporates model uncertainty to guide the selection of the next sampling point. Mathematically, EI quantifies the expected improvement at a given point by combining both the predicted mean and the uncertainty $\hat{\sigma}(m)$.

$$\mathrm{EI}(m) = (\hat{y}_{\mathrm{best}} - \hat{y}(m))\Phi\left(\frac{\hat{y}_{\mathrm{best}} - \hat{y}(m)}{\hat{\sigma}(m)}\right) + \hat{\sigma}(m)\phi\left(\frac{\hat{y}_{\mathrm{best}} - \hat{y}(m)}{\hat{\sigma}(m)}\right) \tag{3}$$

Where $\hat{y}_{\mathrm{best}}$ is the best observed material so far for a specific task, $\hat{y}(m)$ is the GP's

predicted mean at point m, and $\hat{\sigma}(m)$ represents the uncertainty (standard deviation) at point m. Here, $\Phi(\cdot)$ is the cumulative distribution function, and $\phi(\cdot)$ is the probability density function of the standard normal distribution.

The Upper Confidence Bound (UCB) is another commonly used acquisition function that prioritizes exploration by considering uncertainty more explicitly in its formulation. Unlike EI, which balances exploration and exploitation, UCB emphasizes areas with higher uncertainty, making it particularly useful in the early stages of optimization when gathering more information is critical. The UCB acquisition function selects points that maximize a linear combination of the GP's predicted mean $\hat{y}(m)$ and a multiple of the uncertainty $\hat{\sigma}(m)$, encouraging exploration of regions where the model is less certain.

$$\mathrm{UCB}(m) = \hat{y}(m) + \kappa\hat{\sigma}(m) \tag{4}$$

Here, κ is a tunable parameter that controls the balance between exploration and exploitation. A larger κ value encourages more exploration by giving greater weight to the uncertainty term $\hat{\sigma}(m)$, while a smaller κ value focuses more on exploitation by prioritizing the GP's predicted mean $\hat{y}(m)$. UCB is particularly effective in problems where the search space is large, and gaining more information about uncertain regions is crucial for finding the global optimum. In some cases, using a hybrid acquisition strategy that combines both exploitation and exploration, such as alternating between EI and UCB, can enhance performance by balancing the need to refine predictions in known regions while still exploring uncertain areas. This approach is particularly useful in scenarios where both gathering new information and honing in on optimal solutions are crucial.

1.5 Labeling

In practice, the CoRE-2019 and QMOF datasets provide precomputed simulations for CO_2 adsorption and electronic properties of MOFs, respectively. Consequently, we retrieve band

gap or CO_2 uptake capacity data directly from these datasets, eliminating the need for additional costly molecular simulations. This approach allows us to prioritize optimizing the selection process, leveraging existing simulation results as proxies for otherwise resource-intensive calculations.

2 Further Analysis on FABO

2.1 BO with Random Feature Selection

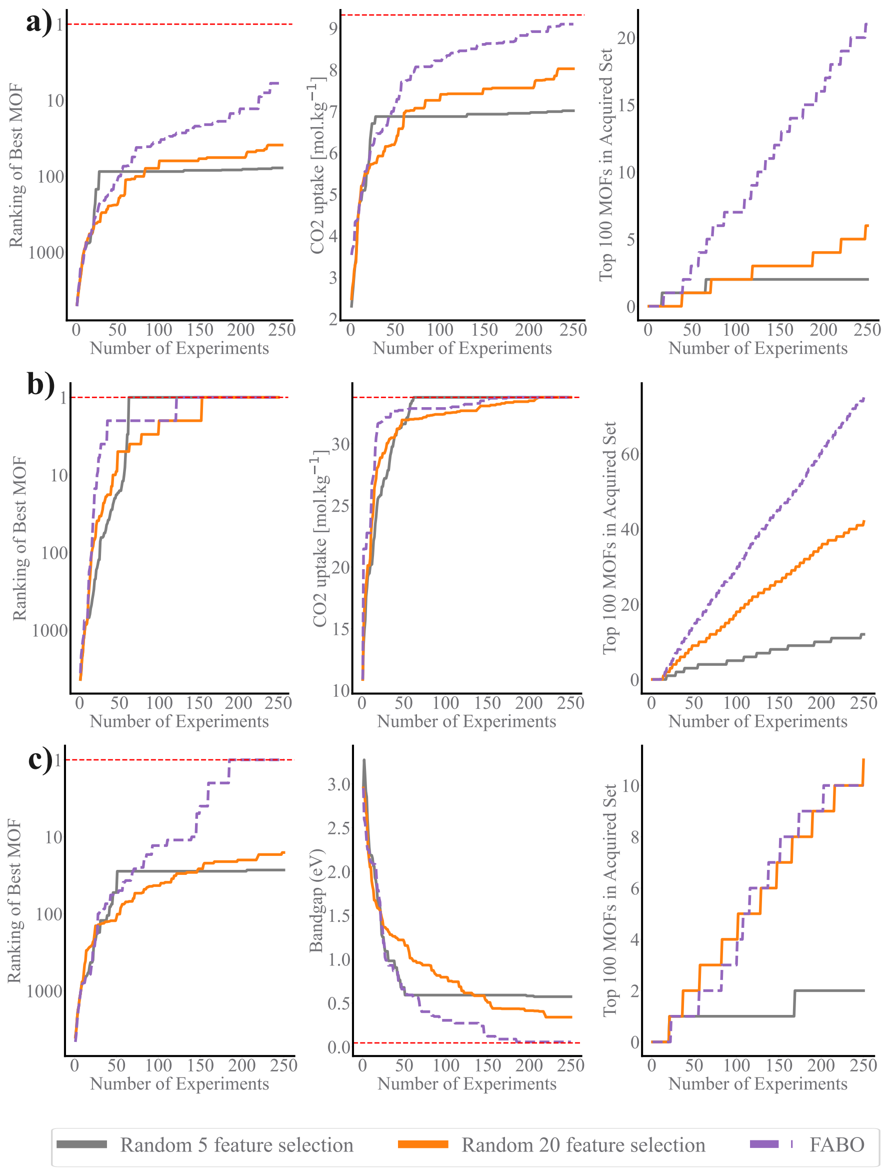

Figure S. 1| Search efficiency curves for FABO, illustrating performance against BO campaigns with random feature selection. (a) CO_2 uptake at low pressure, (b) CO_2 uptake at high pressure, and (c) band gap. The quality of the acquired set of MOFs is shown in three panels: (left) the highest rank relative to the entire dataset; (middle) the optimum value of the objective function; and (right) the number of top 100 MOFs (based on the property of interest) included in acquired MOF set.

2.2 BO with Features Selected from Labeled Dataset

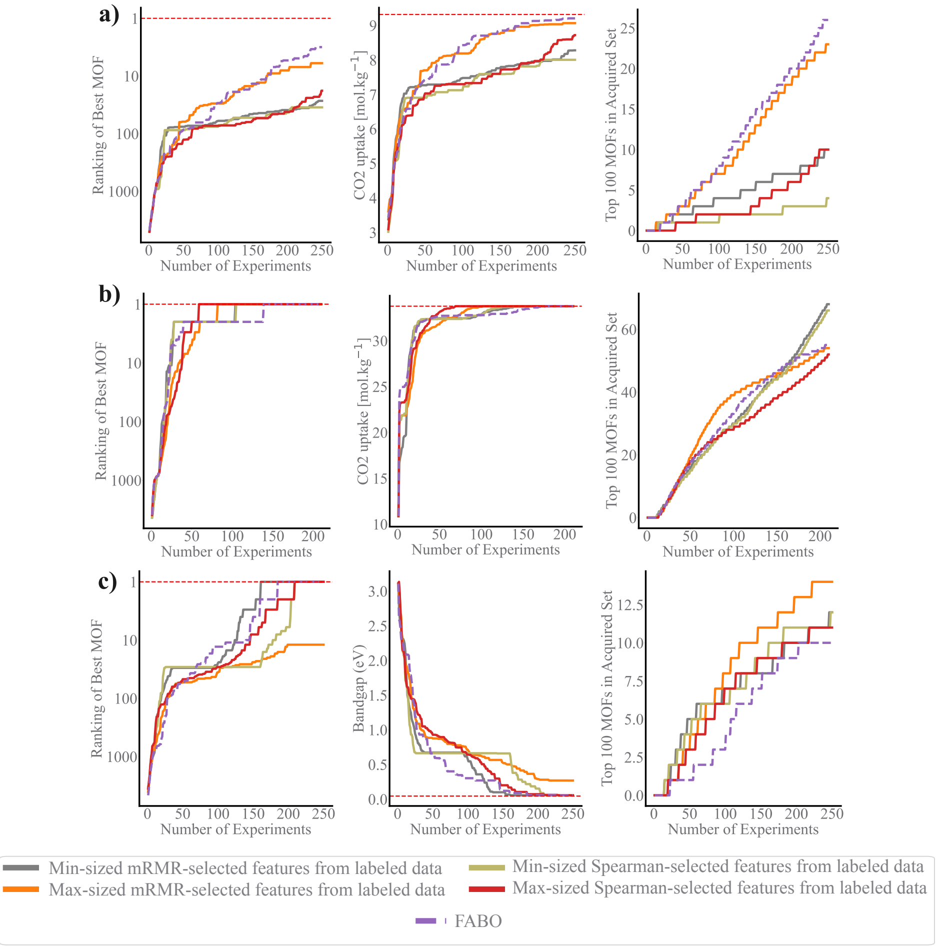

Figure S. 2| Search efficiency curves for FABO, illustrating performance in comparison to BO campaigns with two feature set sizes selected from a labeled dataset using the relevance criterion and Spearman ranking methods. (a) CO_2 uptake at low pressure, (b) CO_2 uptake at high pressure, and (c) band gap. The quality of the acquired set of MOFs is shown in three panels: (left) the highest rank relative to the entire dataset; (middle) the optimum value of the objective function; and (right) the number of top 100 MOFs (based on the property of interest) included in acquired MOF set. For CO uptake, the lower and upper feature counts are 5 and 40, respectively, while for band gap optimization, they are 5 and 20.

2.3 FABO with Different Feature Selection Method

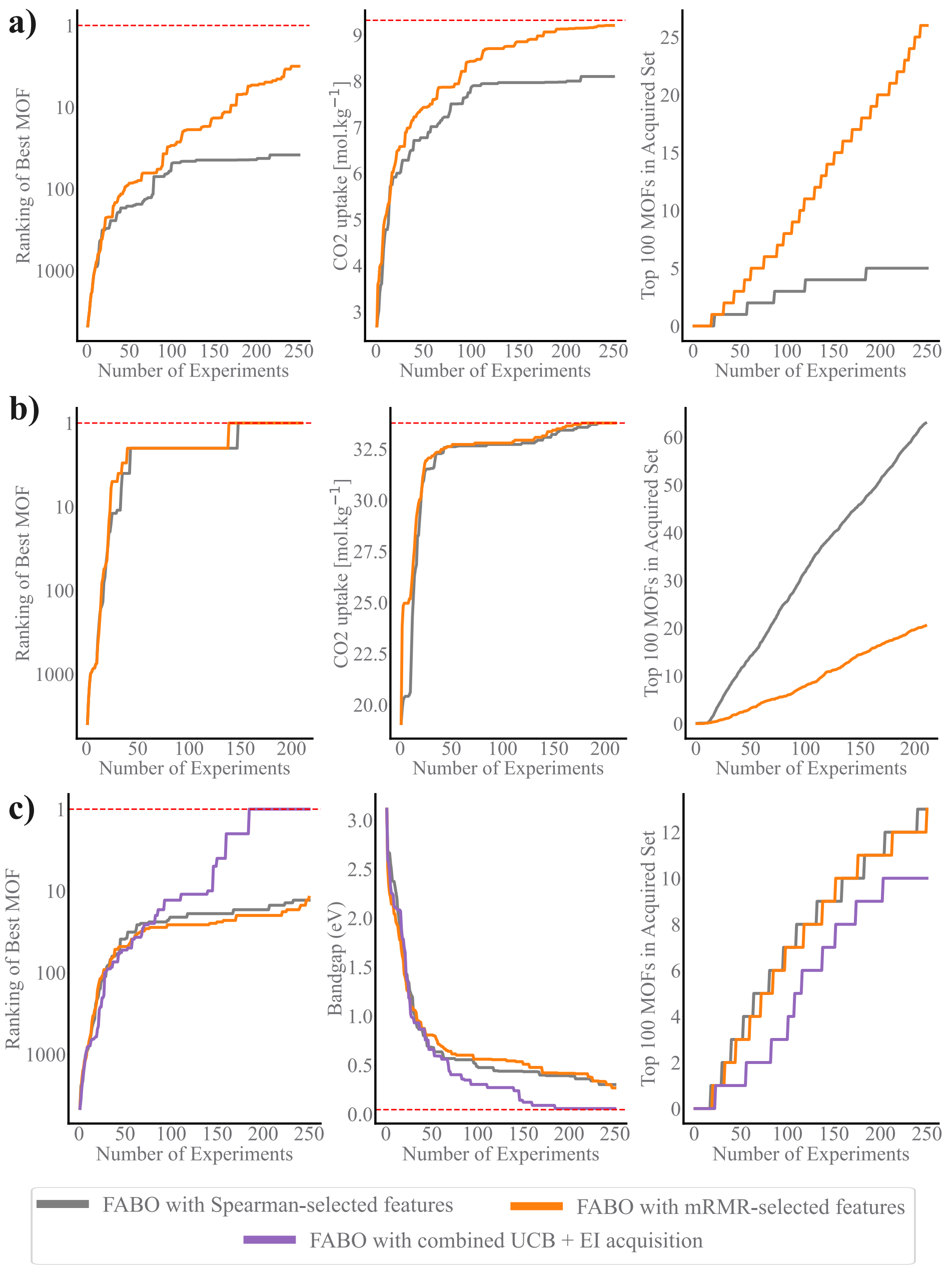

Figure S. 3| Search efficiency curves for FABO operated by different feature selection methods (a) CO_2 uptake at low pressure, (b) CO_2 uptake at high pressure, and (c) band gap. The quality of the acquired set of MOFs is shown in three panels: (left) the highest rank relative to the entire dataset; (middle) the optimum value of the objective function; and (right) the number of top 100 MOFs (based on the property of interest) included in acquired MOF set.

2.4 Benchmarking FABO against BO with random forest as surrogate model

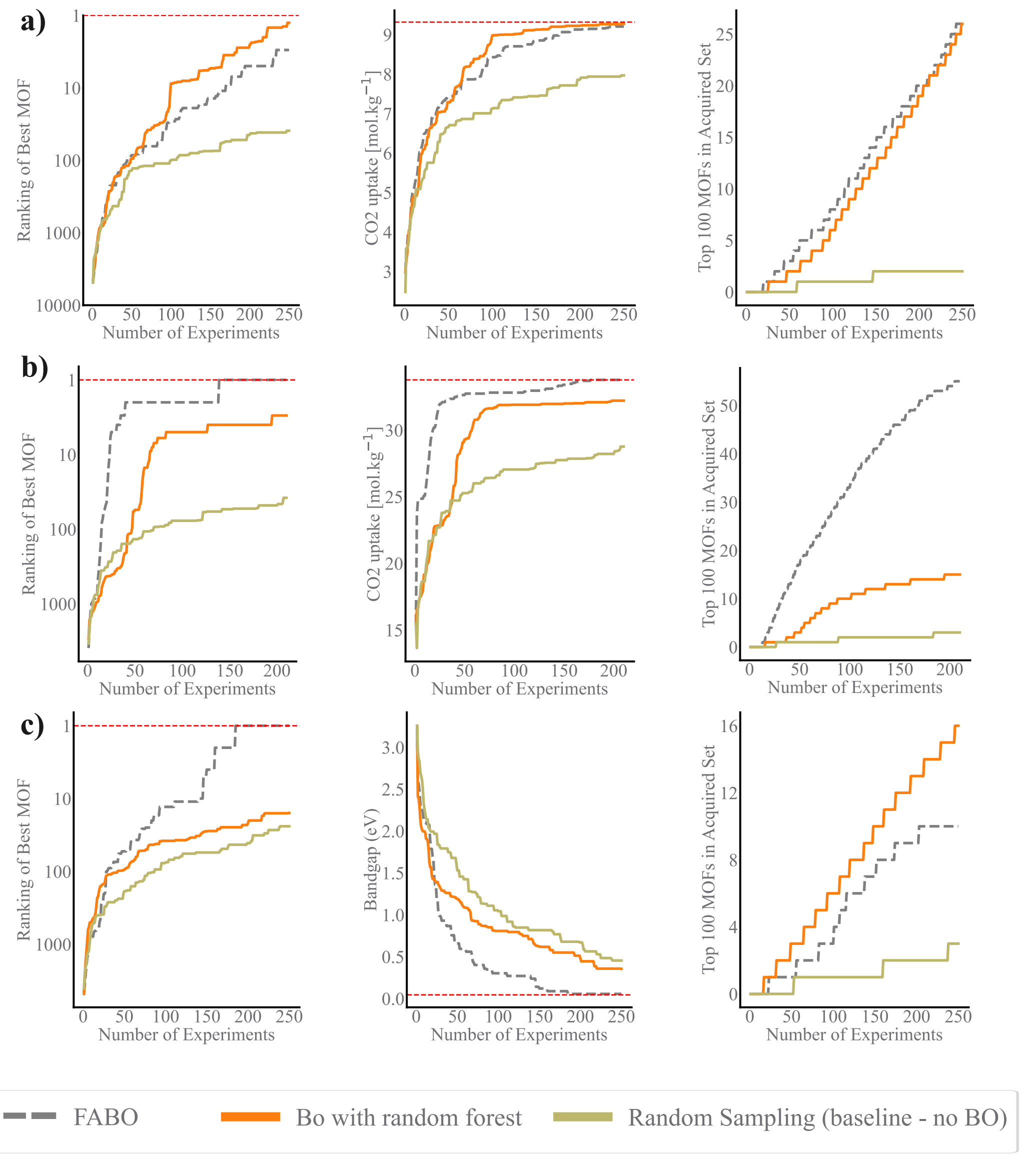

Figure S. 4| Search efficiency curves for FABO, illustrating performance in comparison to BO campaign with random forest as surrogate model (a) CO_2 uptake at low pressure, (b) CO_2 uptake at high pressure, and (c) band gap. The quality of the acquired set of MOFs is shown in three panels: (left) the highest rank relative to the entire dataset; (middle) the optimum value of the objective function; and (right) the number of top 100 MOFs (based on the property of interest) included in acquired MOF set.

2.5 Random Embedding Bayesian Optimization (REMBO)

Previous studies suggested using stochastic random search, as uniform sampling from the feature space can densely cover low-dimensional subspaces without prior knowledge of which dimensions are important.[1,2] Building on this idea, Wang et al. proposed Random Embedding Bayesian Optimization (REMBO), which combines randomization with Bayesian optimization.[3] Specifically, REMBO reduces the dimensionality of the optimization problem by mapping the original high-dimensional feature space ($\mathbb{R}^D$) to a lower-dimensional embedding ($\mathbb{R}^d$) using a random projection matrix $A \in \mathbb{R}^{d\times D}$. Each high-dimensional point $x \in \mathbb{R}^D$ is represented in the low-dimensional space as:

$$z = Ax \tag{5}$$

where $z \in \mathbb{R}^d$ is the lower-dimensional representation. Bayesian optimization is then performed in this reduced space, and the objective function in the high-dimensional space is evaluated by mapping points back using the projection. This method leverages the assumption that the optimal solution lies within a linear subspace of the original feature space, allowing efficient optimization in reduced dimensions.

While REMBO performs well in the CO_2 uptake optimization task at high pressure, it does not outperform FABO and fails to find the best material in the other optimization tasks. This disparity can be attributed to key limitations in REMBO. First, REMBO relies on random projection matrices to reduce dimensionality, which inherently assumes that the optimal solution lies within a linear subspace of the original feature space. However, when features are independent and each carries unique value, such linear combinations may fail to preserve the critical information needed for effective optimization. Second, REMBO lacks a mechanism to adaptively identify or prioritize the most relevant feature dimensions, instead depending on the prior assumption that low effective dimensionality exists. In contrast, FABO dynamically adapts to the data and systematically identifies the most informative

features.

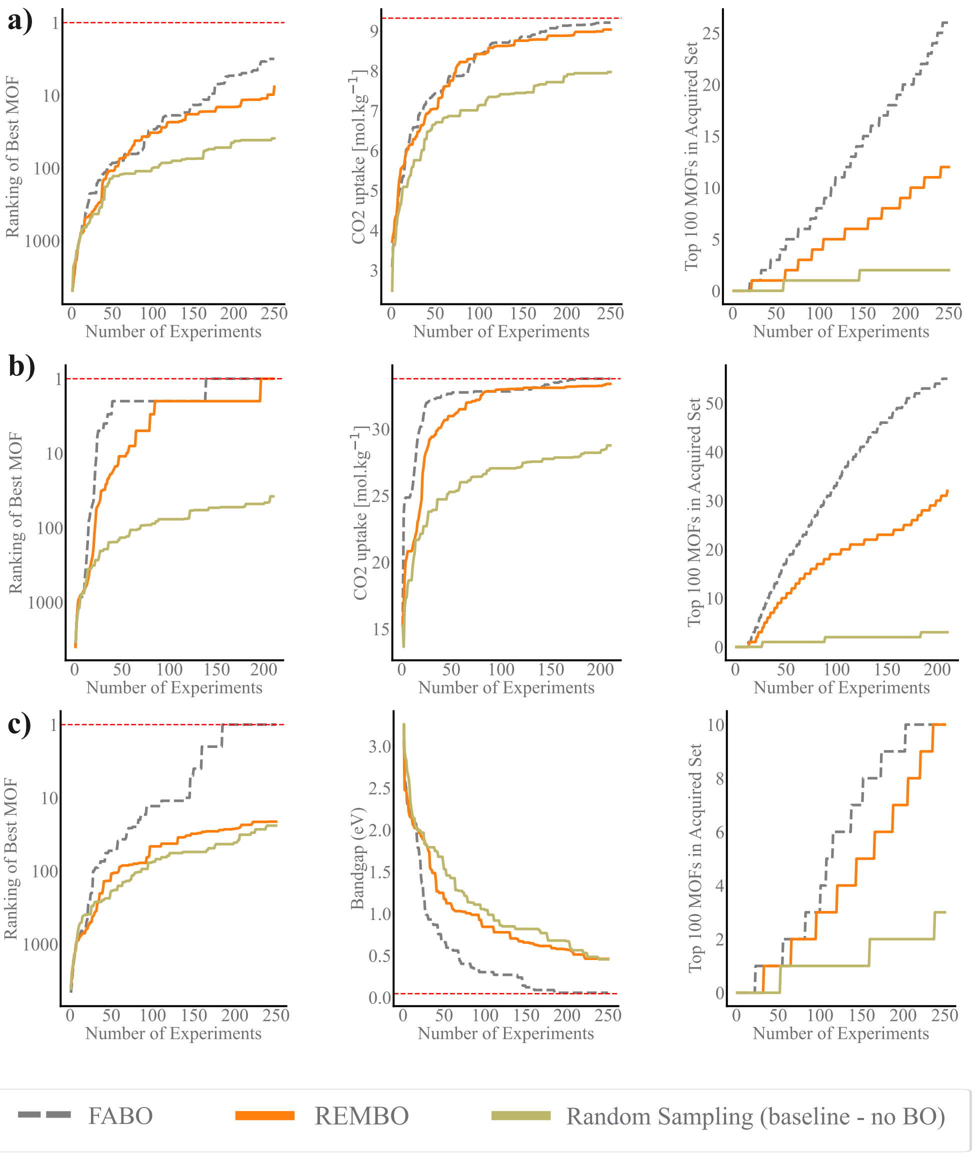

Figure S. 5| Search efficiency curves for FABO, illustrating performance in comparison to Random Embedding Bayesian Optimization (REMBO) (a) CO_2 uptake at low pressure, (b) CO_2 uptake at high pressure, and (c) band gap. The quality of the acquired set of MOFs is shown in three panels: (left) the highest rank relative to the entire dataset; (middle) the optimum value of the objective function; and (right) the number of top 100 MOFs (based on the property of interest) included in acquired MOF set.

2.6 Benchmarking FABO on Molecular Datasets: CHEMBL and Delaney

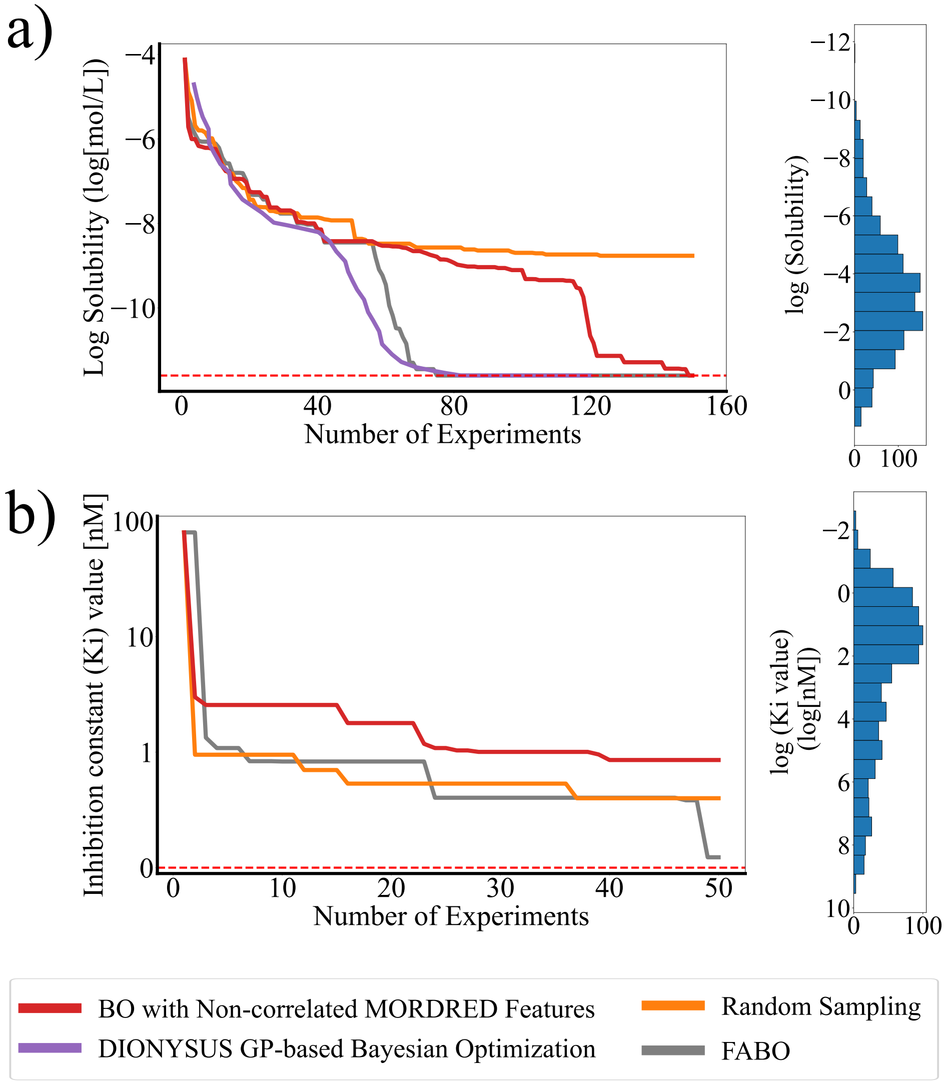

Figure S. 6| FABO benchmarking on molecular datasets (a) Delaney dataset (predicting solubility) and (b) CHEMBL KI 2034 dataset (predicting inhibition constant). In both sub figures, Bayesian Optimization campaign is run using the full feature set generated by the MORDRED Python package, with only zero-variance and highly correlated features (correlation $>$ 0.9) excluded. For the Delaney dataset, FABO is additionally benchmarked against DIONYSUS, a BO model developed by Tom et al., which adjusts the search space dimension via an RBF kernel length parameter to optimize the process.

2.7 Comparative Summary of Material Representation Approaches in Bayesian optimization

Table 1: Comparison of available approaches for identifying the optimal material representation in Bayesian optimization. The FABO achieves superior performance across all cases. The DIONYSUS method shows limited accuracy in CO_2 uptake optimization at high pressure and band gap minimization. Feature selection on fully labeled datasets performs well for mature projects involving MOFs, though it is not applicable to early-stage material discovery, where labeled data is unavailable. Transfer learning is beneficial in specific cases, such as band gap optimization, where a similar, well-characterized property exists. Feature selection based on expert intuition is prone to errors and may be affected by bias.

**Dataset**	**Random**	**Expert Intuition**	**Transfer Learning**	**Feature Selection**	**DIONYSUS**	**FABO**
CO_2 High pressure	✗	✓	✗	✓	✗	✓
CO_2 Low Pressure	✗	✗	✗	✓	✓	✓
Band gap	✗	✗	✓	✓	✗	✓
Practical for new discovery	✓	✓	✗	✗	✓	✓
Bias free	✓	✗	✗	✗	✓	✓
Water Solubility	✗	✗	✗	✓	✓	✓

2.8 Benchmarking FABO on Molecular Datasets: CHEMBL and Delaney

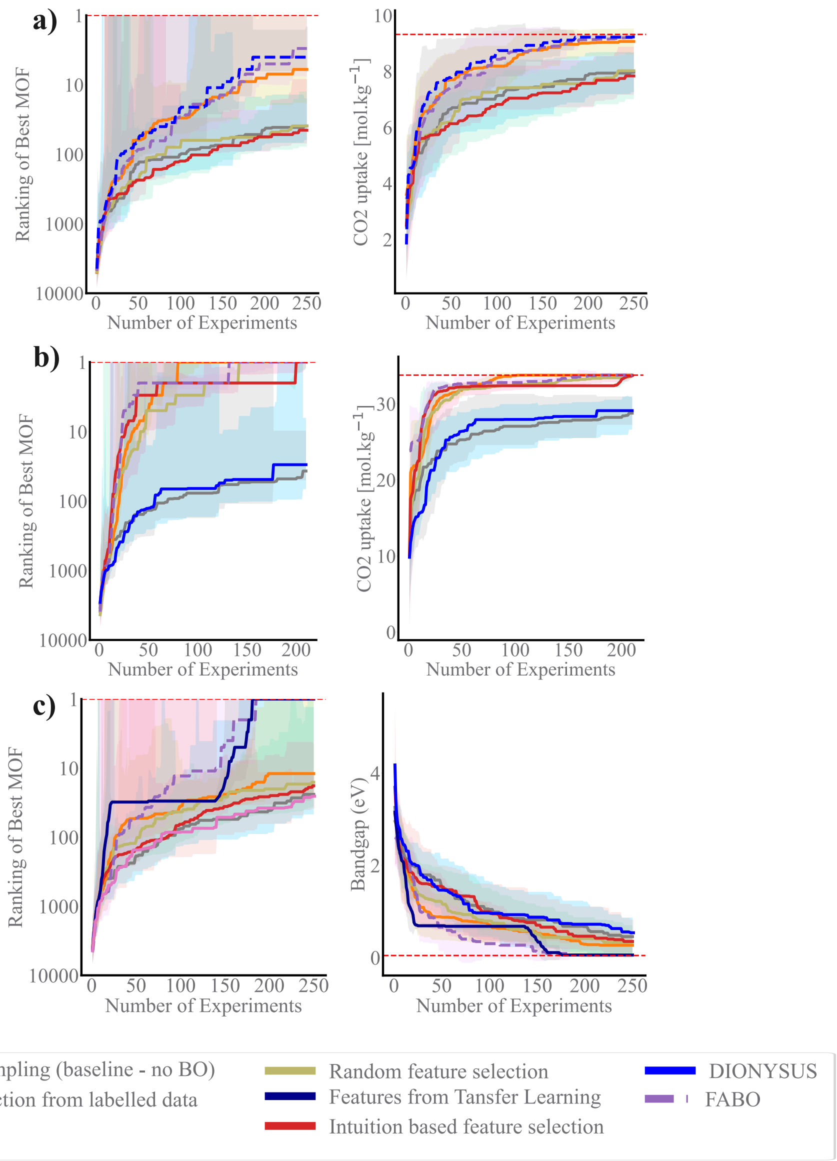

Figure S. 7| Search efficiency curves for different representation methods, including uncertainty. Results show the average across 20 trials for each method, evaluated using two performance metrics: best rank and best value. The shaded area indicates the standard deviation of results: (a) CO_2 uptake at low pressure, (b) CO_2 uptake at high pressure, and (c) bandgap.

3 Compute resources

The experiments were conducted on a personal workstation equipped with the following resources:

- **System RAM:** 32 GB

The runtime for each experimental run, conducted over 250 iterations in the FABO framework, varied depending on the type of feature selector used in the FABO framework and the dataset being processed:

- **Feature Selector: Spearman**
  - **band gap Optimization:** Average runtime of 9 minutes.
  - **Low-Pressure CO_2 Uptake:** Average runtime of 20 minutes.
  - **High-Pressure CO_2 Uptake:** Average runtime of 30 minutes.
- **Feature Selector: mRMR**
  - The runtime for mRMR is approximately double that of Spearman ranking, making it 18 minutes for band gap optimization, 40 minutes for low-pressure CO_2 uptake, and 60 minutes for high-pressure CO_2 uptake.

References

(1) Bergstra, J.; Bengio, Y. Random search for hyper-parameter optimization. *Journal of machine learning research* **2012**, *13*.

(2) Carpentier, A.; Munos, R. Bandit theory meets compressed sensing for high dimensional stochastic linear bandit. Artificial Intelligence and Statistics. 2012; pp 190–198.

(3) de Freitas, N.; Wang, Z. Bayesian Optimization in High Dimensions via Random Embeddings. **2013**,
